# New Insights into Sprout Production from Melon (*Cucumis melo* L. var. *reticulatus*) Seeds as By-Product of Fruit Processing

**DOI:** 10.3390/plants14131896

**Published:** 2025-06-20

**Authors:** Angelica Galieni, Beatrice Falcinelli, Fabio Stagnari, Eleonora Oliva, Federico Fanti, Maria Chiara Lorenzetti, Paolo Benincasa

**Affiliations:** 1 Research Centre for Vegetable and Ornamental Crops, Council for Agricultural Research and Economics [CREA-OF], 63077 Monsampolo del Tronto, Italy; angelica.galieni@crea.gov.it; 2 Department of Agricultural, Food and Environmental Sciences, University of Perugia, 06125 Perugia, Italy; chiara.lorenzetti@unipg.it (M.C.L.); paolo.benincasa@unipg.it (P.B.); 3 Department of Bioscience and Technology for Agriculture Food and Environment, Campus Universitario di Coste Sant’Agostino, University of Teramo, 64100 Teramo, Italy; fstagnari@unite.it (F.S.); eoliva@unite.it (E.O.); ffanti@unite.it (F.F.)

**Keywords:** flavonoids, germination, hydroxybenzoic acids, hydroxycinnamic acids, polyphenol

## Abstract

Melon is a valuable crop that generates significant by-products during consumption and processing. Among these, seeds are rich in phenolic compounds and might be used to produce sprouts with increased content of these bioactive substances. This study evaluated phenolic compounds (PhCs) in sprouts of two melon cultivars, Thales and SV9424ML, obtained from seeds having different germination speeds, thus harvested at 6 and 14 days after sowing (DAS). A factorial combination of cultivar and harvest time was tested in a completely randomized design with four replicates. Thales produced more ready-to-eat sprouts at 6 DAS than SV9424ML (64.0% vs. 46.7%). Sprouting significantly increased total PhCs content, particularly flavonoids, with Thales showing higher values than SV9424ML (50.2 vs. 32.6 mg kg^−1^ DW). Phenolic profiles significantly varied among cultivars and harvests. Sprouts at 6 DAS had more total hydroxybenzoic acids and flavonoids, while 14 DAS sprouts were richer in hydroxycinnamic acids. Significant differences between harvest dates were observed in the concentrations of protocatechuic, vanillic (VanA), *p*-coumaric (*p*-CouA), ferulic (FerA) acids, and orientin (Ori) for Thales, and of VanA, *p*-CouA, FerA, and Ori for SV9424ML. Results are encouraging, but future investigations are essential to understand whether these sprouts can be suitable for fresh consumption, food supplements, or phytochemical extraction.

## 1. Introduction

The generation of large amounts of fruit by-products from the fruit-processing industry represents a significant global challenge [1,2]. Fruit-processing by-products typically comprise 25–60% of the fruit’s weight, primarily consisting of skins, with lesser amounts of pulp and seeds [1]. Traditionally such by-products are used for livestock feed, compost, and, only more recently, for bioenergy production; nevertheless, since they are rich in vitamins, minerals, fiber, oils, and bioactive compounds, they are also a valuable opportunity for biorefinery processes in the pharmaceutical, cosmetic, and food industries [3,4,5].

Seeds, in particular, can be consumed directly or after germination as a source of primary and secondary metabolites and antioxidants [6]. Additionally, they hold the potential for producing edible sprouts allowing an increase in phytochemical content while reducing antinutrients [7]. Notably, sprouts derived from seeds of pomegranate, olive, and *Citrus* species (see the literature cited in [7]) are examples of sprouts obtained from food industry by-products that have been found to show higher phenolic content compared to seeds. By analogy, the potential use of by-products from vegetable species could represent a promising approach, though it remains largely unexplored.

With this regard, melon (*Cucumis melo* L.), a globally consumed and economically valuable fruit, generates significant amounts of by-products during both fresh consumption and industrial processing, with peels and seeds comprising 25–44% and 3.4–7.0% of the fruit’s weight, respectively [1]. Although recent studies have examined the potential utilization of melon by-products [4,5] this area remains relatively understudied and, to the best of our knowledge, there is no study on the use of melon seeds for producing edible sprouts. Melon seeds boast a rich nutritional profile, containing protein, fat, fiber, and various bioactive compounds, such as polyphenols, organic acids, and lignans [2,8,9,10]. In particular, phenolic compounds are worth investigating, for their well-known benefits on human health [8,10]. Nevertheless, the composition and germination performance of melon seeds exhibit significant variability among botanical varieties and cultivars [11]. Moreover, since most cultivated varieties are F1 hybrids, the seeds obtained from fruits represent the F2 generation, which implies segregation and thus different germination and growth performances among offspring individuals. This variability is further exacerbated by environmental variability during pre- and post-harvesting issues [11]. For instance, germination rates may vary among and within plants from which the seeds come, as affected by the timing of development and position of fruits on the same plant [12] and by harvest time and post-harvest storage.

On these bases, this study aimed at obtaining edible sprouts from the seeds discarded from fruits of two different melon (*Cucumis melo* L. var. *reticulatus*) cultivars, investigating germination performances and the accumulation of phenolic compounds during sprouting. Additionally, we deepened the study on the phenolic content of sprouts as affected by a different germination speed within the same seed lot, which caused an 8-day difference in harvest time to obtain ready-to-eat sprouts (fully expanded cotyledons), providing novel insights into the dynamic processes occurring during seed sprouting.

## 2. Results

### 2.1. Germination and Growth Assessment

**Figure 1:**
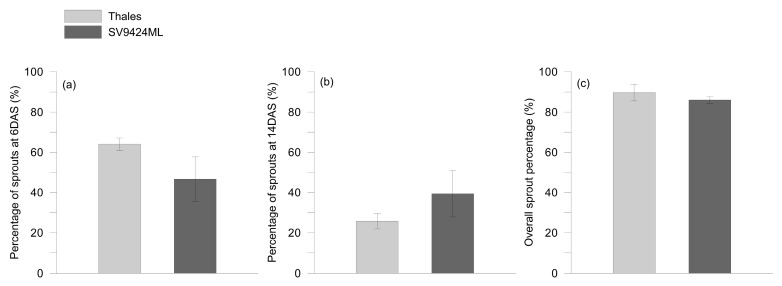
Percentage of sprouts of two melon cultivars (Thales and SV9424ML) harvested at (**a**) 6 and (**b**) 14 days after sowing (DAS), and (**c**) overall. On each date, only sprouts that had reached the ready-to-eat stage (fully expanded cotyledons) were sampled; further details are provided in the text. Data represent means ± standard errors, *n* = 4 independent replicates.

The percentages of sprouts harvested at 6 and 14 days after sowing (DAS) and overall are reported in Figure 1a–c, respectively.

Since, after germination, sprouts appeared not to differ in growth rates, the different percentages of harvested sprouts accounted for a different germination speed within the same seed lot of each cultivar. Although the overall percentage of sprouts harvested did not significantly differ among cultivars (CVs) (Figure 1c), the percentage of sprouts harvested at 6 and 14 DAS clearly varied between Thales and SV9424ML (Figure 1a and 1b, respectively). Thales produced more ready-to-eat sprouts than SV9424ML at 6 DAS (64.0% vs. 46.7%, respectively), while the contrary happened at 14 DAS when the sprouts harvested for SV9424ML were 39.3% against 25.7% for Thales (Figure 1b).

Sprout root and shoot lengths (RL and SL, respectively) and individual sprout fresh weight (FW) did not show any significant difference between CVs (Table 1), whereas the effect of harvests was always significant.

In general, in the sprouts that reached the ready-to-eat stage at 14 DAS, shoot length (SL) was lower by 61%, and root length (RL) was higher by 23% than sprouts harvested at 6 DAS, on average over CVs. This resulted in a significant difference (at *p* < 0.01) in the SL/RL ratio: 0.71 vs. 0.22 on average at 6 and 14 DAS, respectively. On the other hand, SV9424ML showed lower dry matter (DM) % values, particularly at 14 DAS (Table 1).

### 2.2. Phenolic Compounds Variation During Sprouting

**Figure 2:**
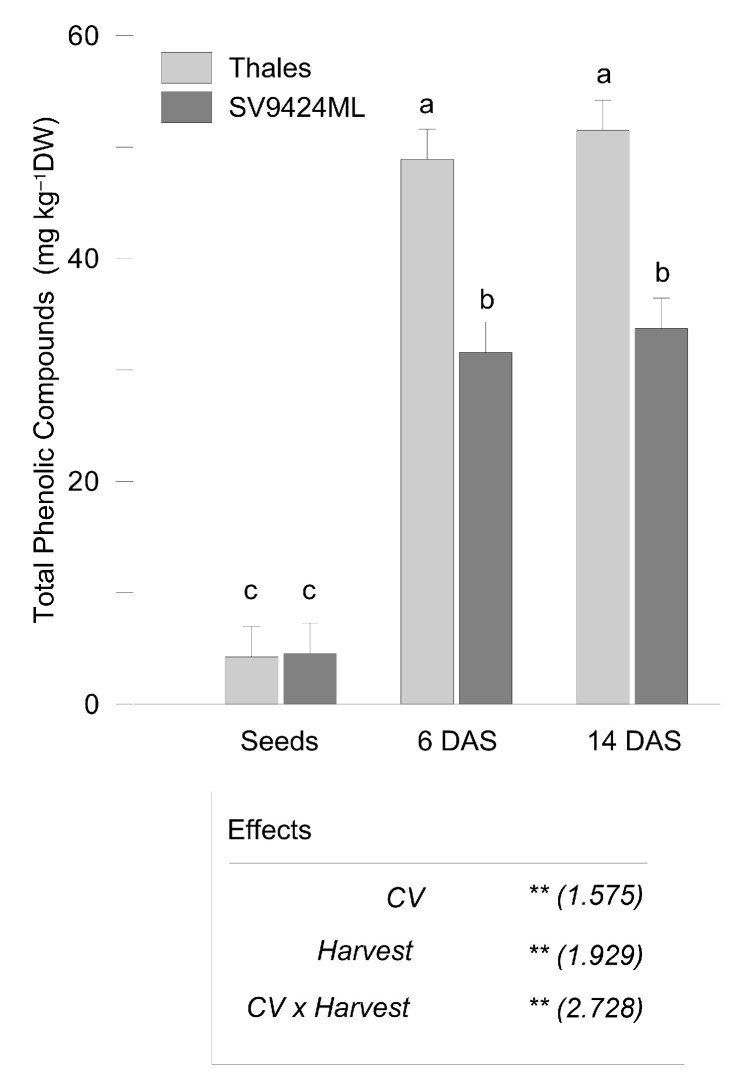
Total Phenolic Compounds (as the sum of all the detected phenolic acids and flavonoids; mg kg^−1^ dry weight, DW) as observed in seeds and sprouts of two melon cultivars (CV; Thales and SV9424ML). Sprouts at the ready-to-eat stage were harvested at two different dates (Harvest; 6 and 14 days after sowing, DAS); further details are provided in the text. Bars represent the standard errors of the differences between means (s.e.d.) of the interaction CV × Harvest. In the boxes, the results of the two-way ANOVA (degrees of freedom: CV, 1; Harvest, 2; CV × Harvest, 2; residues, 18): ** *p* < 0.01); numbers in the brackets represent the s.e.d. Different letters indicate significant differences of the interaction CV × Harvest at *p* < 0.05 (Fisher’s Least Significant Difference, LSD).

The total content of the investigated phenolic compounds (PhCs) (on a DW basis) significantly increased with sprouting, with no significant differences between sprouts harvested at 6 and 14 DAS (9.2- vs. 9.8-fold increase compared to seeds, respectively, averaged across CVs) (Figure 2). Thales sprouts were characterized by higher PhCs (50.17 and 32.63 mg kg^−1^ DW, for Thales and SV9424ML, respectively, on average over harvests).

**Figure 3:**
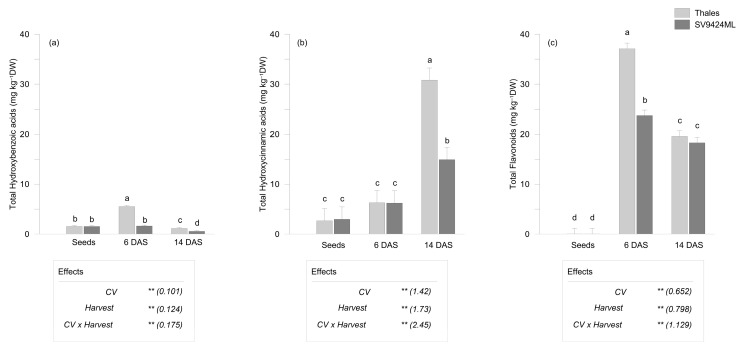
Phenolic compounds (mg kg^−1^ dry weight, DW) as observed in seeds and sprouts of two melon cultivars (CV; Thales and SV9424ML). Sprouts at the ready-to-eat stage were harvested at two different dates (Harvest; 6 and 14 days after sowing, DAS); further details are provided in the text. (**a**) Total hydroxybenzoic acids (as the sum of 4-hydroxybenzoic acid, protocatechuic acid, gallic acid, vanillic acid, syringic acid); (**b**) total hydroxycinnamic acid (as the sum of caffeic acid, chlorogenic acid, *p*-coumaric acid, ferulic acid, trans-cinnamic acid); (**c**) total flavonoids (as the sum of apigenin, luteolin, diosmetin, orientin, rutin, myricetin, naringenin). Bars represent the standard errors of the differences between means (s.e.d.) of the interaction CV × Harvest. In the boxes, the results of the two-way ANOVA (degrees of freedom: CV, 1; Harvest, 2; CV × Harvest, 2; residues, 18): ** *p* < 0.01); numbers in the brackets represent the s.e.d. Different letters indicate significant differences of the interaction CV × Harvest at *p* < 0.05 (Fisher’s Least Significant Difference, LSD).

The variation among the different classes of PhCs depended on the cultivar, harvest date, and their interaction. Specifically, based on LC-MS/MS results, total hydroxybenzoic acids exhibited a 1.4-fold increase (Figure 3a), total hydroxycinnamic acids a 5.2-fold increase (Figure 3b), and total flavonoids an extraordinary 614-fold increase (Figure 3c) compared to seeds, both averaged across CVs and harvests.

Notably, the harvest date, which is the result of germination speed, significantly influenced the phenolic composition of melon sprouts. Sprouts harvested at 6 DAS had higher levels of total hydroxybenzoic acids and total flavonoids (+76% and +38%, respectively, compared to those harvested at 14 DAS, averaged across CVs), while sprouts harvested at 14 DAS had a significantly higher proportion of total hydroxycinnamic acids (+73% compared to those harvested at 6 DAS, averaged across CVs). At 14 DAS, a significantly lower content of total hydroxybenzoic acids compared to seeds was also observed (1.541 and 0.852 mg kg^−1^ DW, in seeds and sprouts harvested at 14 DAS, respectively, on average over CVs) (Figure 3a).

Differences in PhCs between sprouts that were ready-to-eat at 6 and at 14 DAS had the same trends in the two CVs (decrease for hydroxybenzoic acids, increase for hydroxycinnamic acids, and decrease for flavonoids) (Figure 3a–c).

A shift in the profile of phenolic acids was observed between seeds and sprouts: 4-hydroxybenzoic acid (4-OHBenzA) and gallic acid (GalA) were present in seeds but became undetectable after sprouting, and the same was observed for chlorogenic acid (ChA), which was detected only in seeds regardless of the cultivar (Table 2). Flavonoids registered differences even more pronounced, with flavanone naringenin (Nar) being the only compound detected in both seeds and sprouts (Table 3).

Thales generally exhibited significantly higher levels than SV9424ML for the investigated hydroxybenzoic acids (Table 2). Vanillic acid (VanA) was the most abundant, representing 59% and 90% of the total hydroxybenzoic acids found in the sprouts of Thales and SV9424ML, respectively, on average over harvests. Its levels increased from seeds to sprouts harvested at 6 DAS and then significantly decreased for sprouts harvested at 14 DAS. Protocatechuic acid (ProtA) in Thales was lower for ready-to-eat sprouts at 14 DAS than for ready-to-eat sprouts at 6 DAS (Table 2).

The increase in hydroxycinnamic acids with sprouting was primarily due to significant increases in *p*-coumaric acid (*p*-CouA) (76% and 61% of the total hydroxycinnamic acids found in the sprouts of Thales and SV9424ML, respectively) and ferulic acid (FerA) (22% and 37% of the total hydroxycinnamic acids found in the sprouts of Thales and SV9424ML, respectively). The *p*-CouA was also involved in the observed differences in total hydroxycinnamic acids between CV at 14 DAS (18.3-fold and 6.4-fold increases compared to seeds in Thales and SV9424ML, respectively) (Table 2 and Figure 3a).

Among the flavonoids, flavones were the most abundant class in both cultivars. Thales exhibited significantly higher levels than SV9424ML (total flavonoids: 28.3 vs. 21.0 mg kg^−1^ DW, respectively, averaged over harvests—in Tables, values are reported as mg kg^−1^ DW × 10^2^, to improve formatting). Moreover, regardless of CV, the overall flavonoid trends were strongly influenced by the variation in the contents of orientin (Ori) (72% and 78% of the total flavones found in the sprouts of Thales and SV9424ML, respectively) and diosmetin (Dios) (19% and 14% of the total flavones found in the sprouts of Thales and SV9424ML, respectively), with luteolin (Lut) playing a lesser role. Lut and Dios had higher levels in ready-to-eat sprouts at 6 DAS for Thales, while SV9424ML had higher levels in ready-to-eat sprouts at 14 DAS (Table 3).

## 3. Discussion

Sprouting is well acknowledged to induce significant biochemical and nutritional modifications, such as an increase in the phytochemical content with respect to ungerminated seeds [7,13]. Various species have been studied for this purpose, including recently introduced ones [14]. Utilizing seeds that are by-products of processing and fresh consumption is particularly promising. In our study, we investigated the potential use of waste melon seeds in producing edible sprouts.

Initially, we focused on the germination performance (Figure 1). We registered a significant intra-seed-lot variability in the germination speed. This variability likely resulted from the combined effects of (i) the use of open-pollinated seeds from HF1 plants, (ii) inherent differences in germination performance of seeds coming from different plants, fruit positions, and order of fruit set on the mother plants [12,15], (iii) different seed maturity on the same fruit [16], and (iv) the direct use of waste seeds, without any prior storage period [17]. Not surprisingly, genotype influenced germination speed, with Thales exhibiting a higher percentage of ready-to-eat sprouts obtained at 6 DAS as compared to SV9424ML.

It should be noted that in sprout production, good and uniform germination as well as high seedling vigor are needed for the proper setup of the production process. Moreover, sprouts are entirely consumed, including rootlets [13]. Consequently, seeds are germinated using highly available, cost-effective, and microbiologically safe sprouting media, while prolonging germination times could have implications from a microbiological point of view. To overcome staggered germination, it could be useful to (i) select cultivars with minimal intrinsic variability, (ii) implement automated tools to assess seed vigor [18] allowing for the creation of separate seed lots based on germination speed, and (iii) leverage the positive effects of storage and/or seed priming techniques—including non-thermal technologies [19,20]—on melon seed germination performances.

The observed variability in the germination speed implied that sprouts reached the ready-to-eat stage at different dates (Figure 1 and Table 1), and this induced us to test a hypothesis that, to our knowledge, has not been considered in the literature, namely that the germination speed and thus the different harvest times of sprouts might somehow be linked to differences in the sprout phytochemical content.

Indeed, we obtained interesting findings about the phenolic composition responses of melon sprouts. Firstly, we observed a general increase in total PhCs (as the sum of all the individual free phenolic acids and flavonoids detected) with sprouting (Figure 2), regardless of the harvest date but depending on CV, likely associated with the marked upregulation of the enzymatic activity—particularly those involved in cell wall degradation and phenolic biosynthesis [7].

Secondly, sprouting modified heavily the accumulation of single phenolic compounds in melon (Figure 3). Regardless of CV, phenolic acids (Table 2) represented the most representative class of PhCs in seeds followed by flavonoids, confirming previous studies [21] and disagreeing with others [6]. However, we found some differences from the literature in terms of phenolic composition, probably attributable to the extraction procedures [22]. In the sprouts, as compared to seeds, the flavonoid content (Table 3) increased significantly (about 60% of the total PhCs), in particular Ori and Dios. Comparing our data with existing literature is challenging due to the lack of studies on melon sprouts. Araújo et al. [23] reported an increase in the total flavonoid and phenolic compounds in germinated seeds of four melon varieties. However, despite using waste melon seeds, their study focused primarily on germinated seeds rather than on the production of edible sprouts; the profiles of the PhCs were not reported, and the germinated seeds were harvested within 96 h. Finding references on other species within the *Cucurbitaceae* family is also difficult and sometimes contradictory, depending on genetic structure, production conditions, agricultural and climatic factors, and the analytical and extraction techniques used [24].

Thirdly, the harvest date, a direct consequence of the readiness of seeds to germinate, seemed to strongly modify the phenolic compound profile: excluding hydroxybenzoic acids—much lower in their overall content—sprouts that reached the ready-to-eat sprouts at 6 DAS, i.e., those coming from more vigorous seeds, were richer in flavonoids but lower in hydroxycinnamic acids, with no substantial differences between cultivars (Figure 3). We hypothesized two possible explanations for this evidence that deserve further investigation. On one hand, since the biochemical composition tends to differ among aerial and above-ground tissues—as observed, for example, in Chinese kale sprouts [25] and durum wheat [26]—the differences found in terms of phytochemical profiles can be attributable to the different shoot-to-root ratios of sprouts harvested at 6 and 14 DAS (Table 1). Differences among roots and shoots also emerged in terms of glucosinolate content and diversity from a comprehensive analysis of 29 species, mainly belonging to cultivated *Brassica* species and varieties, likely due to variations in biosynthesis and turnover regulations, as well as organ-specific expression patterns of several transcription factors [27]. On the other hand, the phenolic content in plant cells can depend on the up- or down-regulation of key enzymes in the biosynthetic pathways, as well as on their degradation rates mediated by oxidases and peroxidases, although establishing a link between these phenomena is challenging [28]. In the phenolic pathway, i.e., the shikimic acid pathway, *p*-coumaryl-CoA [synthesized from L-phenylalanine through the production of intermediate compounds such as cinnamic acid and *p*-coumaric acid] serves as an activated intermediate for both the synthesis of hydroxycinnamic acids and flavonoids. Cinnamate 3-hydroxylase (C3H) is the final key limiting enzyme for hydroxycinnamic acids synthesis while chalcone synthase (CHS) is involved in the activation of the flavonoid branch pathway [28]. In our study, we speculate that there might have been a differential expression of the C3H and/or CHS enzymes depending on the germination speed, as it is plausible that the sprouts harvested at 14 DAS may have also undergone specific stressful conditions. For example, in tomatoes, the C3H activity—and consequently hydroxycinnamic acid production—is overexpressed under salt stress while being inhibited under heat [28]. Additionally, *p*-CouA is the precursor of Nar [with the addition of three molecules of malonic acid and with the action of CHS and chalcone isomerase] which is actually lower than the other flavonoids and thus suggesting that the high content of *p*-CouA recorded in sprouts at 14 DAS (Table 2) would be employed in another metabolic pathway. *p*-CouA and FerA are involved in the formation of monolignols [after polymerization with their alcohols, i.e., 4-coumaric alcohol and coniferyl alcohol, respectively] and then in the biosynthesis of lignin, which plays an important role against biotic and abiotic stress [29,30]. On the other hand, the high content of Ori (i.e., the most represented flavones with high antioxidant properties and related numerous health benefits [31]) (Table 3) recorded mainly in sprouts at 6 DAS would represent a strategy of more vigorous seeds to face the stress occurring during the germination process. It is not surprising that the extent of these described variations was influenced by the genetic material of seeds (i.e., F2) and by the cultivar too.

Lastly, the greatest contribution among the hydroxycinnamic acids was provided by *p*-coumaric and ferulic acids (Table 2), while flavones were the main class found among flavonoids (Table 3). For the well-known effects of these compounds as antioxidants, specific reviews are referenced (see, for example, Sun and Shahrajabian) [32].

## 4. Materials and Methods

### 4.1. Plant Material and Sprouting Conditions

Seeds of two melon HF1 cultivars (CV)—Thales (Syngenta^®^ Sementi Orticole Italia, Syngenta Italia S.p.A., Milan, Italy) and SV9424ML (Seminis, Bayer Vegetables Italia, Bayer Group, Milan, Italy)—were obtained from commercially mature fruits harvested on 28 July 2021, from a crop cultivated by the Top Melon farm (Top Melon s.r.l., Pantalla, Perugia province, Italy) in a field of the mid-Tiber Valley plain, Central Italy. The crop had been grown in an open field, with only plastic mulch and with low tunnels along the furrow to limit pollinator circulation until blooming, allowing more contemporary pollination of pistillate flowers and thus a more contemporary ripening of fruit, as described in Benincasa et al. [33] Nutrient fertilization, irrigation, and protection against pests and diseases were guaranteed according to the best management practices adopted in that cultivation area. After harvest, fruits were immediately hand processed, separating seeds from fruits and washing and rinsing them with tap water to remove pulp residues. Finally, an additional washing with distilled water was performed. The number of seeds needed for any replicate in any experiment was taken randomly from the seed bulk obtained. Some seeds from each CV were frozen in liquid nitrogen and stored at −80 °C until chemical analysis. A preliminary germination test was performed with two replicates of 100 seeds for each of the two cultivars. Since we observed a certain variability in the germination speed within the seed lot of each CV, we decided to plan the sprouting experiment including, as a variable, two harvest dates (Harvest) differing by about one week, to harvest, on each date, only sprouts that had reached the ready-to-eat stage, assumed to be at fully expanded cotyledons. To obtain sprouts, the yielded seeds were sown in plastic trays containing sterile paper laid over sterile cotton wetted with distilled water. The trays were covered by a drilled top to maintain air circulation while preventing dehydration. The trays were incubated in a growth chamber at 20 °C in a light:dark regime of 12:12 h. Treatments (i.e., CV × Harvest) were laid down according to a completely randomized design with four replicates (trays). The two harvest dates then resulted in 6 and 14 days after sowing (6 DAS and 14 DAS, respectively).

At each harvest date, fresh and dry weights were measured on a subsample of 20 sprouts per replicate, and the dry matter content (DM, %) was calculated; sprouts were also characterized for their shoot and root lengths (SL and RL, respectively). The rest of the sampled sprouts of each replicate were lyophilized, finely homogenized, and stored at −20 °C until the analysis.

### 4.2. Chemicals

All the chemicals were of analytical reagent grade; all polyphenols reported in Table S1 were purchased from Sigma-Aldrich (St. Louis, MO, USA). The phenolic compounds (PhCs) stock solutions were prepared in MeOH at a concentration of 1.0 × 10^−^^2^ mol L^−1^ and stored at −20 °C in the dark. All solvents for PhC analysis, such as MeOH, ACN, H_2_O, formic acid, and acetic acid, were purchased from VWR (West Chester, PA, USA); all solvents were UHPLC grade.

### 4.3. UPLC-ESI-MS/MS Analysis

Quantitative analysis of PhCs was carried out on both melon seeds and sprouts. All the PhCs listed in Section 4.2 were identified; additionally, the total amount of PhCs was reported as the sum of all the individual detected PhCs.

An Acquity H-Class chromatographic system (Waters, Milford, MA, USA) coupled with a Qtrap4500 mass spectrometer (Sciex, Toronto, ON, Canada) was used according to Oliva et al. [34], with slight modifications. Briefly, the samples were ground under liquid nitrogen and freeze-dried. Subsequently, 0.1 g of each sample was weighed and extracted with 1 mL of a MeOH:H_2_O solution (70:30 *v*:*v*) with ultrasonic-assisted extraction (UAE) for 30 min at room temperature and centrifuged at 10,000 rpm for 10 min at 4 °C. The supernatant was picked up and the pellet was extracted again under the same conditions. The collected extract was dried by SpeedVac Vacuum Concentrator system (Thermo Fischer, Waltham, MA, USA) and the pellet was resuspended with 1 mL of phosphate buffer (50 mM) H_2_O at pH 3:MeOH (90:10 *v*:*v*) for the clean-up step. The purification phase was carried out by Solid Phase Extraction (SPE) with Strata XL cartridge (330 mg, 1 mL) from Phenomenex (Torrance, CA, USA), then analyzed by UPLC-ESI-MS/MS in Multiple Reaction Monitoring (MRM) acquisition modes operating in negative ionization. For chromatographic separation, an Excel 2 C18-PFP 2.0 µm (100 × 2.1 mm) column was employed; H_2_O 1% acetic acid (phase A) and ACN (phase B) were used as mobile phases. The column operated at a flow rate of 0.4 mL/min. The column oven was set at 40 °C. For detection, the MRM acquisition was used; for each PhC, at least two MRM transitions were monitored, and each of them was carefully tuned by the injection of the correlated analytical standard. All MRM transition parameters are reported in Table S1. Data collection and processing were performed with Analyst 1.7.3 software and quantification with Multiquant 3.0.3 software, both from Sciex.

### 4.4. Statistical Analysis

Data were analyzed by two-way ANOVA and the effects of genotype (CV), harvest date (Harvest), and their interaction (CV × Harvest) were tested. ANOVA assumptions were verified through graphical methods. When ANOVA revealed significant differences, means separation was performed through Fisher’s least significant difference test (LSD) at *p* < 0.05. The R statistical environment was used to analyze data [35].

## 5. Conclusions

This study highlights the potential of melon seed by-products for sprouting applications. Melon seeds demonstrated good germination performance; however, intra-seed-lot variability in germination speed required two separate harvests. This variability is typical of F1 hybrid offspring, particularly when derived from immature fruits, harvested from different plant positions, and subjected to varying post-harvest storage conditions.

Focusing on quality traits, melon sprouts exhibited higher total phenolic content than ungerminated seeds, especially in flavonoids. The phenolic profile was significantly influenced by the cultivar and time required to obtain ready-to-eat sprouts—which correlates with germination speed—with the Thales cultivar showing higher phytochemical content, particularly in early harvests. These findings indicate that germination speed (more generally, seed vigor) and cultivar selection could be key factors in optimizing the nutritional quality of melon sprouts.

Sprouting melon seeds appears an effective strategy for valorizing this by-product, allowing a high yield of phenolic compounds. Further research is needed to assess taste, potential antinutrient content, and the suitability of these sprouts for fresh consumption or phytochemical extraction, as well as to elucidate the biochemical pathways involved in sprout formation and phytochemical synthesis.

## Figures and Tables

**Table 1:** Root length (RL, mm sprout^−1^), shoot length (SL, mm sprout^−1^), fresh weight (FW, mg sprout^−1^), and dry matter (DM, %) of sprouts of two melon cultivars (CV; Thales and SV9424ML). Sprouts at the ready-to-eat stage were harvested at two different dates (harvest: 6 and 14 days after sowing, DAS); further details are provided in the text. Data represent means ± standard errors of n = 4 independent replicates; means separation was performed using Fisher’s Least Significant Difference (LSD) at *p* < 0.05.

Cultivar	Harvest	RL (mm Sprout^−1^)	SL (mm Sprout^−1^)	FW (mg Sprout^−1^)	DM (%)
Thales					
Sprouts at 6 DAS		101.3 ± 8.44	64.8 ± 2.52	240.2 ± 5.19	6.44 ± 0.025
Sprouts at 14 DAS		126.2 ± 13.27	28.3 ± 1.09	180.4 ± 10.43	6.80 ± 0.001
Overall		113.8	46.6	210.3	6.62
SV9424ML					
Sprouts at 6 DAS		90.3 ± 2.18	68.7 ± 1.91	201.6 ± 13.73	6.31 ± 0.247
Sprouts at 14 DAS		109.7 ± 7.30	23.0 ± 1.59	186.3 ± 2.40	5.82 ± 0.181
Overall		100.0	45.8	193.9	6.07
F-test					
CV		n.s.	n.s.	n.s.	** (0.153)
Harvest		* (8.74)	** (1.85)	** (9.08)	n.s.
CV × Harvest		n.s.	* (2.61)	* (12.84)	* (0.217)
LSD					
CV		19.03	4.03	19.79	0.33
Harvest		19.03	4.03	19.79	0.33
CV × Harvest		26.91	5.70	27.98	0.47

Two-way ANOVA: n.s. not-significant; * *p* < 0.05; ** *p* < 0.01. Degree of freedoms: cultivar (CV), 1; harvest dates (Harvest), 1; CV × Harvest, 1; residues, 12. In brackets: standard errors of the differences between means (s.e.d.).

**Table 2:** Phenolic acids observed in seeds (0 days after sowing, DAS) and sprouts of two melon cultivars (CV; Thales and SV9424ML). Sprouts at the ready-to-eat stage were harvested at two different dates (Harvest; 6 and 14 days after sowing, DAS); further details are provided in the text. Phenolic acids are listed as (i) hydroxybenzoic acids (4-hydroxybenzoic acid, 4-OHBenzA; protocatechuic acid, ProtA; gallic acid, GalA; vanillic acid, VanA; syringic acid, SyrA) and (ii) hydroxycinnamic acids (caffeic acid, CafA; chlorogenic acid, ChA; *p*-coumaric acid, *p*-CouA; ferulic acid, FerA; *trans*-cinnamic acid, trans-CinA). Data represent means ± standard errors of *n* = 4 independent replicates; means separation was performed using Fisher’s Least Significant Difference (LSD) at *p* < 0.05.

Effects	Hydroxybenzoic Acids (mg kg^−1^ DW × 10^2^)					Hydroxycinnamic Acids (mg kg^−1^ DW ×10^2^)				
	4-OHBenzA	ProtA	GalA	VanA	SyrA	CafA	ChA	*p*-CouA	FerA	*trans*-CinA
Thales										
Seeds	54.6 ± 0.49	30.5 ± 1.14	2.5 ± 0.09	36.6 ± 0.66	29.9 ± 0.71	30.5 ± 0.53	8.6 ± 0.26	133.5 ± 2.04	40.6 ± 0.57	51.6 ± 1.58
Sprouts at 6 DAS	n.d.	235.6 ± 20.71	n.d.	305.8 ± 3.68	6.5 ± 0.58	7.9 ± 0.10	n.d.	359.7 ± 8.17	216.0 ± 12.83	44.4 ± 4.35
Sprouts at 14 DAS	n.d.	25.0 ± 6.36	n.d.	83.2 ± 1.29	5.9 ± 0.72	13.3 ± 2.23	n.d.	2448.7 ± 322.23	582.6 ± 108.00	34.9 ± 4.90
Overall	--	97.0	--	141.9	14.1	17.2	--	980.6	279.7	43.6
SV9424ML										
Seeds	48.0 ± 2.51	37.9 ± 0.32	2.4 ± 0.08	35.1 ± 0.96	30.7 ± 0.87	40.2 ± 0.63	7.8 ± 0.23	145.5 ± 4.36	41.4 ± 0083	61.2 ± 3.81
Sprouts at 6 DAS	n.d.	1.02 ± 0.09	n.d.	154.8 ± 19.03	7.6 ± 0.97	7.6 ± 0.58	n.d.	350.5 ± 10.67	242.0 ± 13.22	20.9 ± 1.13
Sprouts at 14 DAS	n.d.	0.27 ± 0.02	n.d.	43.3 ± 1.19	12.7 ± 0.06	12.3 ± 1.96	n.d.	932.0 ± 82.56	530.4 ± 36.18	14.8 ± 1.25
Overall	--	13.1	--	77.7	17.0	20.1	--	476.0	271.2	32.3
F-test										
CV	--	** (7.23)	--	** (6.50)	** (0.58)	* (1.05)	--	** (110.98)	n.s.	** (2.64)
Harvest	--	** (8.86)	--	** (7.96)	** (0.71)	** (1.28)	--	** (135.92)	** (47.11)	** (3.23)
CV × Harvest	--	** (12.52)	--	** (11.26)	** (1.01)	** (1.81)	--	** (192.23)	n.s.	** (4.57)
LSD										
CV	--	15.19	--	13.65	1.22	2.20	--	233.16	80.80	5.55
Harvest	--	18.60	--	16.72	1.50	2.69	--	285.57	98.96	6.80
CV × Harvest	--	26.31	--	23.65	2.12	3.81	--	403.85	139.96	9.61

Two-way ANOVA: n.s. not-significant; * *p* < 0.05; ** *p* < 0.01. Degrees of freedom: cultivar (CV), 1; harvest dates (Harvest), 2; CV × Harvest, 2; residues, 18. In brackets: standard errors of the differences between means (s.e.d.). n.d.: not detectable. Since phenolic acids’ profile varied among seeds and sprouts, the two-way ANOVA was applied only to phenolic compounds present in all stages.

**Table 3:** Flavonoids observed in seeds (0 days after sowing, DAS) and sprouts of two melon cultivars (CV; Thales and SV9424ML). Sprouts at the ready-to-eat stage were harvested at two different dates (Harvest; 6 and 14 days after sowing, DAS); further details are provided in the text. Flavonoids are listed as (i) flavones (apigenin, Api; luteolin, Lut; diosmetin, Dios; orientin, Ori), (ii) flavonols (rutin, Rut; myricetin, Myr), and (iii) flavanones (naringenin, Nar). Data represent means ± standard errors of *n* = 4 independent replicates; means separation was performed using Fisher’s Least Significant Difference (LSD) at *p* < 0.05.

Effects	Flavones (mg kg^−1^ DW × 10^2^)				Flavonols (mg kg^−1^ DW × 10^2^)		Flavanones (mg kg^−1^ DW × 10^2^)
	Api	Lut	Dios	Ori	Rut	Myr	Nar
Thales							
Seeds	n.d.	n.d.	n.d.	n.d.	2.6 ± 0.35	1.4 ± 0.05	0.36 ± 0.036
Sprouts at 6 DAS	6.8 ± 0.40	261.4 ± 13.71	607.1 ± 48.12	2797.1 ± 64.58	n.d.	n.d.	38.2 ± 2.44
Sprouts at 14 DAS	5.3 ± 0.28	154.7 ± 18.86	462.7 ± 53.02	1299.6 ± 78.59	n.d.	n.d.	32.2 ± 4.52
Overall	--	--	--	--	--	--	23.6
SV9424ML							
Seeds	n.d.	n.d.	n.d.	n.d.	2.3 ± 0.08	1.3 ± 0.02	0.12 ± 0.022
Sprouts at 6 DAS	4.3 ± 0.49	56.9 ± 7.24	257.4 ± 25.33	2026.6 ± 58.81	n.d.	n.d.	26.0 ± 1.57
Sprouts at 14 DAS	4.9 ± 0.09	200.5 ± 8.58	347.1 ± 19.82	1238.7 ± 58.42	n.d.	n.d.	33.7 ± 4.80
Overall	--	--	--	--	--	--	19.9
F-test							
CV	--	--	--	--	--	--	n.s.
Harvest	--	--	--	--	--	--	** (2.94)
CV × Harvest	--	--	--	--	--	--	n.s.
LSD							
CV	--	--	--	--	--	--	5.04
Harvest	--	--	--	--	--	--	6.18
CV × Harvest	--	--	--	--	--	--	8.73

Two-way ANOVA: n.s. not-significant; ** *p* < 0.01. Degrees of freedom: cultivar (CV), 1; harvest dates (Harvest), 2; CV × Harvest, 2; residues, 18. In brackets: standard errors of the differences between means (s.e.d.). n.d.: not detectable. The two-way ANOVA was applied only to flavanones, which were present in all stages.

## Data Availability

The original contributions presented in this study are included in the article. Further inquiries can be directed to the corresponding author.

## Supplementary Materials

The following supporting information can be downloaded at https://www.mdpi.com/article/10.3390/plants14131896/s1.
