# Supplementary material for: New Insights into Sprout Production from Melon (Cucumis melo L. var. reticulatus) Seeds as By-Product of Fruit Processing
Source: Plants (Basel). 2025 Jun 20;14(13):1896. doi: 10.3390/plants14131896 (PMC12252063; doi:10.3390/plants14131896)
Supplement: Supplementary file 1 [file plants-14-01896-s001.zip › plants-3662774-supplementary.pdf]

Table S1. MRM transition of the UPLC-MS/MS method; for all transition precursor mass (Q1), fragment mass (Q3), declustering potential (DP), entrance potential (EP), collision energy (CE) and collision cell exit potential (CXP) are reported.

Analyte	Q1(m/z)	DP(eV)	EP(eV)	Q3(m/z)	CE(eV)	CXP(eV)
Tyrosol	136.8	-30	-9	105.8	-22	-7
				106.0	-22	-4
OH-Benzoic Acid	137.0	-30	-8	92.9	-16	-6
				66.0	-30	-8
Trans-Cinnamic Acid	147.0	-37	-8	102.9	-16	-4
				77.2	-30	-6
Protocatechuic Acid	153.0	-35	-12	109.0	-23	-6
				53.0	-34	-4
OH-Tyrosol	153.0	-55	-7	122.9	-21	-9
				104.6	-30	-7
Coumaric Acid	163.0	-70	-5	116.9	-42	-9
				119.0	-20	-9
Vanillic Acid	166.8	-66	-6	107.8	-31	-9
				151.8	-21	-5
Gallic Acid	168.9	-30	-5	124.9	-21	-9
				78.9	-33	-6
Caffeic Acid	178.9	-30	-4	134.9	-22	-4
				107.0	-32	-10
Ferulic Acid	192.9	-43	-11	134.0	-21	-8
				177.7	-18	-6
Syringic Acid	197.0	-20	-11	181.9	-19	-6
				121.0	-22	-8
Sinapic Acid	223.0	-55	-11	163.8	-23	-6
				207.8	-22	-7
Resveratrol	226.8	-90	-10	184.8	-28	-11
				142.8	-37	-11
Apigenin	268.9	-100	-4	117.0	-50	-9
				150.9	-34	-5
Naringenin	271.0	-80	-9	150.9	-25	-7
				118.9	-37	-8
Luteolin	284.9	-100	-8	150.9	-35	-9
				133.0	-44	-4
Kaempferol	284.9	-100	-8	229.0	-35	-7
				184.9	-38	-6
Catechin	289.0	-90	-8	244.9	-24	-9
				108.9	-38	-8
Epicatechin	289.0	-90	-8	244.9	-24	-9
				108.9	-38	-8
Diosmetin	299.0	-70	-6	255.9	-40	-9

				283.9	-31	-10
Ellagic Acid	301.0	-130	-6	283.9	-43	-9
				145.0	-50	-6
Quercetin	300.9	-84	-10	151.0	-30	-6
				178.8	-26	-6
Epigallocatechin	305.0	-80	-6	136.9	-36	-10
				124.9	-29	-9
Caftaric Acid	311.1	-35	-9	179.0	-22	-7
				149.0	-16	-6
Myricetin	317.0	-75	-4	179.1	-29	-6
				150.9	-33	-6
Chlorogenic Acid	353.1	-65	-7	191.1	-24	-6
				160.9	-35	-6
Xanthohumol	353.1	-110	-7	233.0	-29	-5
				233.0	-29	-5
Isoxanthohumol	353.1	-110	-7	233.0	-29	-5
				233.0	-29	-5
Rosmarinic Acid	359.0	-50	-6	161.0	-26	-7
				197.1	-25	-7
Orientin (Luteolin-8-Glucoside)	447.3	-90	-11	327.0	-34	-8
				356.6	-32	-11
Epigallocatechin gallate	457.3	-15	-10	169.2	-20	-11
				125.1	-40	-11
Quercetin-Hexoside	462.9	-110	-5	300.9	-48	-10
				270.9	-58	-10
Hesperidin	609.3	-100	-7	163.9	-75	-9
				301.0	-36	-10
Rutin	609.3	-100	-6	299.7	-50	-10
				270.9	-79	-7
Galangin	268.9	-110	-3	169.0	-39	-11
				171.0	-39	-10
Daidzein	252.9	-110	-4	222.9	-45	-7
				131.9	-50	-4
Isorhamnetin	314.9	-30	-9	300.0	-31	-10
				150.9	-39	-5
Genistein	268.9	-70	-10	132.9	-39	-4
				159.0	-40	-8
Fisetin	284.9	-80	-7	134.8	-29	-4
				121.0	-34	-8
